# Drugging PI3K in cancer: refining targets and therapeutic strategies

**DOI:** 10.1016/j.coph.2015.05.016

**Published:** 2015-08

**Authors:** Timothy A Yap, Lynn Bjerke, Paul A Clarke, Paul Workman

**Affiliations:** 1 Cancer Research UK Cancer Therapeutics Unit, Division of Cancer Therapeutics, The Institute of Cancer Research, 15 Cotswold Road, Sutton, Surrey SM2 5NG, UK; 2 Royal Marsden NHS Foundation Trust, Downs Road, Sutton, Surrey SM2 5PT, UK; 3 Division of Molecular Pathology, The Institute of Cancer Research, 15 Cotswold Road, Sutton, Surrey SM2 5NG, UK

## Abstract

The phosphatidylinositol-3 kinase (PI3K) pathway is one of the most frequently activated pathogenic signalling routes in human cancers, making it a rational and important target for innovative anticancer drug development and precision medicine. The three main classes of PI3K inhibitors currently in clinical testing comprise dual pan-Class I PI3K/mTOR inhibitors, pan-Class I PI3K inhibitors lacking significant mTOR activity and isoform-selective PI3K inhibitors. A major step forward in recent years is the progression of over 30 small molecule PI3K inhibitors into clinical trials and the first regulatory approval of the PI3Kδ inhibitor idelalisib for multiple B-cell malignancies. This review article focuses on the progress made in the discovery and development of novel PI3K inhibitors, with an emphasis on antitumour activity and tolerability profiles for agents that have entered clinical trials. We also discuss the key issues of drug resistance, patient selection approaches and rational targeted combinations. Finally, we envision the future development and use of PI3K inhibitors for the treatment of patients with a range of malignancies.

## Highlights

• PI3K is an important target for innovative anticancer drug development and precision medicine.
• Over 30 small molecule PI3K inhibitors are currently in clinical trial testing.
• These drugs include dual PI3K/mTOR, pan-Class I PI3K and isoform-selective PI3K inhibitors.
• The PI3Kδ inhibitor idelalisib has received FDA approval for the treatment of B-cell malignancies.
• Drug resistance, patient selection and development of targeted combinations remain challenges.

**Current Opinion in Pharmacology** 2015, **23**:98–107

This review comes from a themed issue on **Cancer**

Edited by **Alex N Phipps**

For a complete overview see the Issue and the Editorial

Available online 25th June 2015

**http://dx.doi.org/10.1016/j.coph.2015.05.016**

1471-4892/© 2015 The Authors. Published by Elsevier Ltd. This is an open access article under the CC BY license (http://creativecommons.org/licenses/by/4.0/).

## Introduction

The phosphatidylinositol-3 kinase (PI3K) pathway is one of the most frequently activated pathogenic signalling routes in human cancers, affecting 30–50% of malignancies, making it a rational and important target for innovative anticancer drug development and precision medicine [1, 2]. There are four well-described Class I PI3K isoforms (α, β, δ and γ encoded by *PIK3CA*, *PIK3CB*, *PIK3CD* and *PIK3CG* respectively) which catalyze phosphorylation of phosphoinositides on the 3′ position of the inositol ring, and most importantly, the conversion of PtdIns(4,5) to the second messenger PtdIns(3,4,5) or PIP_3_ — which in turn recruits cytosolic proteins with PIP_3_-binding pleckstrin homology (PH) domains (such as the serine/threonine kinase protein kinase B/AKT), thereby localizing them to the plasma membrane. The Class IA isoforms (α, β and δ) in particular are associated with oncogenesis, cancer progression and multiple hallmarks of malignancy [3^••^].

**Figure 1:**
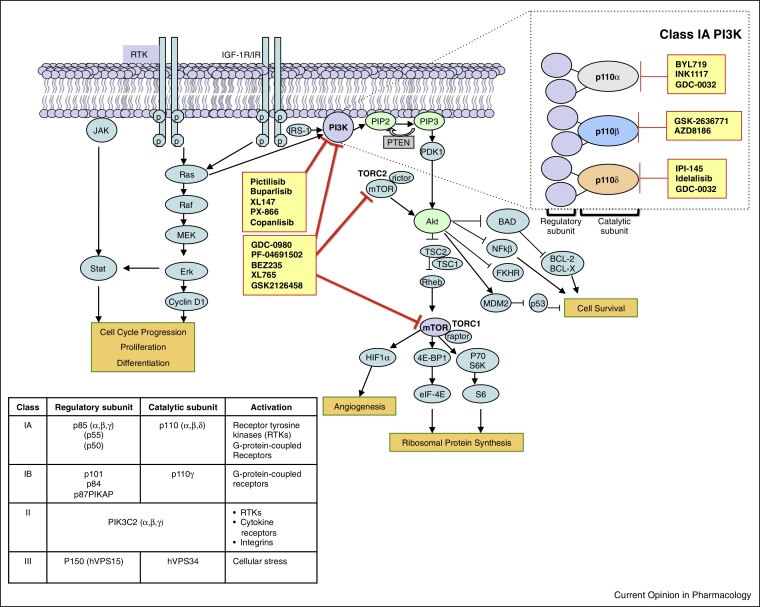
The PI3K pathway with respective PI3K inhibitors. When PI3K is activated, phosphatidylinositol 3,4,5-trisphosphate (PIP3) is generated from phosphatidylinositol 3,4-bisphosphate (PIP2), and recruits AKT to the cell membrane [80, 81]. This leads to a conformational change and phosphorylation of AKT and its subsequent activation. AKT then translocates to the cytoplasm and nucleus, where phosphorylation of various downstream substrates involved in the regulation of multiple cellular functions, including proliferation, survival and growth occurs. The PI3K pathway is one of the most frequently activated signalling pathways in human cancers, affecting 30–50% of tumours, making it a rational target for novel anticancer drug development. The red arrows indicate the respective mechanisms of action of different PI3K inhibitors, which include the dual PI3K/mTOR inhibitors, pan-Class I PI3K inhibitors and isoform-selective PI3K inhibitors. Individual examples of different PI3K inhibitors in clinical testing are shown in the figure. The table in the figure lists the regulatory and catalytic subunits of the respective PI3K classes.

The *PIK3CA* gene, which encodes the p110α catalytic subunit of PI3K, is the most commonly mutated kinase in the human genome [4]. The identification of driver *PIK3CA* mutations through tumour genome sequencing provided the first example of a mutated lipid kinase oncogene [5^••^]. There is also evidence of *PIK3CA* amplification and overexpression in different cancers, as well as numerous other oncogenic abnormalities, including frequent mutation, deletion and loss of expression of the tumour suppressor gene *PTEN* (Figure 1) [6, 7]. Further research is required to delineate the relationship of such aberrations with other oncogenic abnormalities, so as to improve our understanding of potential mechanisms of drug resistance, which may have implications for the development of effective targeted combination regimens.

**Figure 2:**
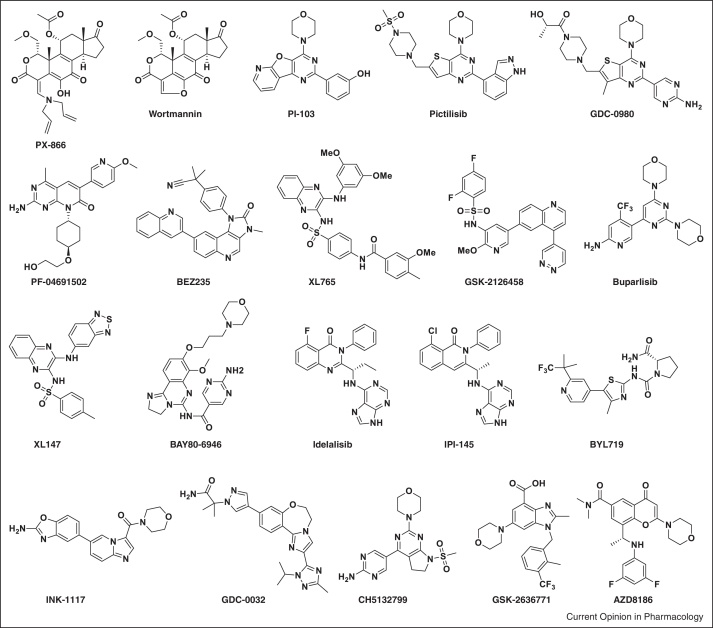
Chemical structures of PI3K inhibitors highlighted in this article.

A major step forward in recent years has been the progression of over 30 small molecule PI3K inhibitors into clinical trials and the first regulatory approval of one such agent, idelalisib (Zydelig, CAL-101; Gilead Sciences) (see Figure 2 for representative chemical structures) [8, 9••, 10••]. This present article focuses on the recent progress made in the discovery and development of novel PI3K inhibitors, with an emphasis on antitumour activity and tolerability profiles for agents that have entered clinical trials. We also discuss the key issues of patient selection, drug resistance and rational targeted combinations. Finally, we envision the future development and use of PI3K inhibitors for the treatment of patients with a range of different malignancies.

## Current status of PI3K inhibitors

The three main classes of PI3K inhibitors currently in clinical testing comprise dual pan-Class I PI3K/mTOR inhibitors, pan-Class I PI3K inhibitors lacking significant mTOR activity and isoform-selective PI3K inhibitors [11]. The vast majority of these drugs are ATP-competitive reversible kinase inhibitors, while PX-866 (Oncothyreon), which is based on the earlier potent natural product but unstable inhibitor wortmannin, is the only irreversible PI3K inhibitor currently in clinical trial testing [12]. An interesting spectrum of varying drug characteristics, with respect to both antitumour activity and tolerability, has been observed across the different PI3K inhibitors. Such findings have influenced drug discovery and development strategies and also regulatory registration approaches with PI3K inhibitors.

Of special note, in July 2014 the US Food and Drug Administration (FDA) approved the first PI3K to be licensed, namely the PI3Kδ inhibitor idelalisib for different B-cell malignancies: as monotherapy for patients with relapsed follicular B-cell non-Hodgkin lymphoma and small lymphocytic lymphoma, and in combination with rituximab for those with relapsed chronic lymphocytic leukemia (CLL) [8, 9••, 10••]. The other classes of PI3K inhibitors are still in early to late clinical trial testing in solid tumours and/or haematological malignancies utilising either monotherapy or combination strategies.

## Tolerabilty profiles of PI3K inhibitors

The safety profiles of all three classes of PI3K inhibitors are now well characterised through the clinical testing of multiple agents. PI3K/mTOR inhibitors include GDC-0980 (Genentech), which is related to the increasingly used chemical tool compound PI-103 [13], PF-04691502 (Pfizer), BEZ235 (Novartis), XL765 (Exelixis/Sanofi-Aventis) and GSK2126458 (GlaxoSmithKline) [11, 14, 15]. These agents share common, dose-dependent drug-related toxicities comprising rash, fatigue, hyperglycemia, as well as gastrointestinal symptoms including nausea, vomiting and diarrhoea. Such effects appear to be on-target, but differences in both frequency and severity of certain adverse events between various agents have been observed. Overall, these side-effects have limited the long-term tolerability of the dual PI3K/mTOR targeting agents.

Inhibitors that target essentially all the Class I PI3 kinases with minimal or no mTOR inhibition include the oral compounds pictilisib (GDC-0941; Genentech), buparlisib (BKM120; Novartis), XL147 (SAR245408; Exelixis/Sanofi) and PX-866, together with the intravenous BAY80-6946 (copanlisib; Bayer) [16, 17, 18, 19, 20]. The toxicities seen with this class of agents are similar, albeit probably less severe to those observed with PI3K/mTOR inhibitors, and include rash, hyperglycaemia, gastrointestinal symptoms and fatigue. For the intravenous inhibitor BAY80-6946, at its maximum tolerated dose, six of seven evaluable patients required insulin treatment for glucose levels above 200 mg/dL, which occurred within 24 h of dosing [21].

Despite the selectivity of the α, δ and γ isoform-specific PI3K inhibitors, drug-related toxicities observed are generally similar to those observed with pan-Class I PI3K inhibitors, including nausea, vomiting, diarrhoea and fatigue. However, hyperglycaemia is frequently observed with PI3Kα inhibitors such as BYL719 and GDC-0032 [22, 23], in contrast to δ and γ isoform-specific PI3K inhibitors for which myelosuppression with neutropenia and raised liver transaminases have been reported, whereas hyperglycaemia is relatively uncommon [8, 9••, 10••].

The disparate effects are consistent with the known biology of PI3K isoforms [11]. However, there is a need to develop better animal models to predict human toxicities with PI3K inhibitors, and in particular to understand any additive versus synergistic toxicities with combination strategies involving these agents.

## Antitumour activity of PI3K inhibitors

### PI3K/mTOR inhibitors

With PI3K/mTOR inhibitors, despite the vertical blockade of the two different crucial nodes along the PI3K signalling pathway, single agent antitumour activity has been modest [11]. For example, only two of 78 patients, one each with bladder and renal cell carcinoma, had RECIST partial responses with GSK2126458 [24]. This may partly be due to the narrow therapeutic window associated with these drugs that limits their dose escalation, or to the unselected populations of patients enrolled into these early phase studies. Several PI3K/mTOR inhibitors are currently being tested in ongoing single agent or combination Phase II studies.

### Pan-Class I PI3K inhibitors

Similarly, despite a favourable pharmacokinetic–pharmacodynamic (PK–PD) profile, including evidence of target engagement by measuring downstream phosphoprotein biomarkers, only modest evidence of single agent antitumour activity has been observed with the oral pan-Class I PI3K inhibitors [16, 17]. Anecdotal examples of antitumour responses to pictilisib include a patient with oncogenic V600E BRAF-mutant melanoma and another with platinum-refractory epithelial ovarian cancer exhibiting both PTEN expression loss and *PIK3CA* amplification [16]. Interestingly, in an expansion cohort of patients with non-Hodgkin's lymphoma treated with BAY80-6946, 5 RECIST partial responses were observed in six evaluable patients, with FDG-PET studies confirming disease regression [21]. A phase II study of BAY80-6946 in patients with non-Hodgkin's lymphoma is ongoing (NCT01660451) [25]. Activity in this setting is likely to be driven by the subnanomolar IC50 potency of BAY80-6946 against PI3Kδ [26].

### Isoform-selective PI3K inhibitors

#### PI3Kδ isoform-specific inhibitors

As discussed, robust single agent clinical activity has been observed with the FDA-approved PI3Kδ inhibitor idelalisib in B-cell malignancies that exhibit lineage-dependency on this isoform. PI3Kδ plays a crucial role in B cell regulation, including proliferation and survival, and demonstrates high expression in leukocytes [27]. The observation that PI3Kδ is especially hyperactivated in different B cell cancers provided strong rationale for developing potent and PI3Kδ-isoform specific inhibitors in such haematological malignancies, rather than solid tumours [28]. This was supported by multiple preclinical studies demonstrating that such a strategy would lead to selective B cell cytotoxicity with minimal effects on other haematopoietic cells [29].

It is likely that its increased specificity for the PI3Kδ isoform, together with reduced hyperglycaemia has enabled the administration of relatively higher doses of idelalisib, leading to enhanced target and pathway suppression, potentially greater than that obtainable with dual PI3K/mTOR inhibitors and pan-Class I PI3K inhibitors. Another promising PI3Kδ isoform-specific PI3K inhibitor is IPI-145 (Infinity Pharmaceuticals) which has also demonstrated impressive results in patients with relapsed haematological cancers, including CLL and lymphoma [30].

#### PI3Kα isoform-specific inhibitors

While there is a general lack of consensus between studies involving *PIK3CA* mutant cell sensitivity to pan-Class I PI3K inhibition [31, 32, 33, 34, 35, 36], recent preclinical studies indicate that *PIK3CA* mutant cancer cells are indeed more sensitive to the p110α-specific inhibitors BYL719 (Novartis) and INK1117 (Millennium) [37, 38] and the ‘p110β-sparing’ p110α inhibitors GDC-0032 (Genentech) and CH5132799 (Chugai Pharmaceutical) [33, 39]. Early phase clinical trials involving the p110α inhibitors BYL719 and GDC-0032 have demonstrated signals of therapeutic activity with RECIST responses in *PIK3CA* mutant solid tumours, providing early proof-of-concept for this targeted monotherapy approach [22, 23].

On the basis of a robust preclinical relationship, only patients with advanced solid tumours harbouring *PIK3CA* aberrations were enrolled onto the phase I trial of p110α-specific BYL719; of 102 patients treated, nine RECIST partial responses (four confirmed) were reported [22]. Similarly, in the first-in-human phase I study of GDC-0032, from 12 patients with *PIK3CA* mutant solid tumours, five objective responses were reported [23]. Four of these *PIK3CA* mutant responders had breast cancer, indicating a potential molecularly selected tumour type for more focused clinical testing in the future. This is especially important since only one further response was observed in the other 22 patients with different advanced solid cancers, which were all confirmed to be *PIK3CA* wildtype.

#### PI3Kβ isoform-specific inhibitors

Preclinical data have indicated that PI3Kβ isoform-specific inhibition is more potently effective in cancer cells with PTEN loss [40, 41], and clinical trials involving PI3Kβ-targeted inhibitors GSK-2636771 (GlaxoSmithKline) [42] and AZD8186 (AstraZeneca) [43] are currently ongoing to explore the safety and antitumour activity in patients with tumours characterised by PTEN deficiency or PI3Kβ aberrations. Interestingly, the phase I trial of GSK2636771 demonstrated PI3K pathway inhibition, but limited antitumour activity in patients with PTEN-deficient tumours [42]. However, early signals of antitumour activity with RECIST responses were observed, suggesting that patients with tumours harbouring concomitant genetic alterations in *PIK3CB* may benefit from PI3Kβ blockade; exploratory genomic analyses of tumour samples from 10 prostate cancer patients revealed a *PIK3CB* L1049R mutation in a tumour specimen from a patient who remained on study for 33 weeks, and an increased tumoural *PIK3CB* gene copy number in a patient who achieved a RECIST partial response.

Recently, important insights have been revealed about potential limitations of α and β isoform-specific PI3K inhibitors. With regards to PTEN loss and p110β dependency, evidence now suggests that concurrent mutations that activate p110α, such as an activated *KRAS* mutation, can cause a context-dependent shift away from PI3Kβ inhibitor sensitivity to PI3Kα inhibitor responsiveness [44]. Furthermore, recent studies demonstrate that cancer cells which are initially dependent upon either PI3Kα or PI3Kβ can overcome isoform-specific inhibitory selection pressures through the upregulation of the alternate class I isoform [45••, 46••]. Crucially, these recent data demonstrate a level of robustness in PI3K signalling, suggesting that pan-Class I PI3K inhibitors or the combination of potent PI3Kα and PI3Kβ inhibitors may ultimately be required to impact different cancers by suppressing or overcoming the upregulation of the non-targeted PI3K isoforms.

## Challenges in patient selection strategies

Moving forward, it will be important to establish clear patient selection criteria using predictive biomarkers of response and resistance for the different classes of PI3K inhibitors. Such patient selection is however complex and multi-factorial, and is likely to be affected by issues of intra-tumoural and inter-tumoural heterogeneity [47] and also by the development of crosstalk and disruption of signalling feedback loops [48]. For example, a recent study demonstrated that the frequent presence of subclonal driver mutations, such as E545K *PIK3CA*, may necessitate the stratification of targeted therapy response according to the percentage of tumour cells in which the driver is identified [49]. Positioning of PI3K gene mutations on the trunk or branches of evolutionary trees is also likely to be important for the extent or duration of response.

Genotype-based sensitivity correlation studies that included PI3K inhibitors have involved the assessment of large cancer cell line panels in high-profile publicly available publications and associated datasets such as the Cancer Genome Project (CGP, Sanger Centre) and the Cancer Cell Line Encyclopaedia (CCLE, Broad Institute) [50, 51]. Overall, large discrepancies were identified between the sensitivity-genotype associations identified by these two data sources, although the DNA sequencing and RNA expression data were found to be concordant [52^••^]. Because of this lack of consensus, it is currently unclear which of these genetic associations are fully robust and further studies are required to resolve this. Note that it is important to consider precise PI3K isoform selectivities and to include multiple different inhibitor chemotypes so as to minimize off-target effects and maximize robustness of findings [53].

Animal models are important to establish the quantitative extent and duration of on-target and pathway inhibition necessary for both biological and therapeutic effects [54]. These data can then be used to correlate preclinical PK–PD profiles to toxicity and efficacy results. For example, preclinical studies of the pan-Class I PI3K inhibitor pictilisib demonstrated that greater than 90% inhibition of AKT phosphorylation over several hours is required for 50% reduction in the number of proliferating cancer cells *in vitro* and subsequent growth arrest in tumour xenografts [20, 55]. In the subsequent phase I trial of pictilisib, PK was dose-proportional, while phosphorylated AKT levels were suppressed >90% in both platelet-rich plasma at 3 h after dosing and in tumour at doses associated with PK (area under curve [AUC]) >20 h μmol/L [16]. Significant increases in plasma insulin and glucose levels, as well as >25% decrease in (18)F-fludeoxyglucose (FDG) uptake by positron emission tomography (PET) in seven of 32 evaluable patients also confirmed target modulation. Despite these promising PK–PD data, single agent antitumour activity was modest, with limited RECIST responses observed [16].

Drug response prediction studies have generally utilised mutation, copy number and gene fusion data to generate a measure of pathway deregulation. This strategy has allowed accurate prospective identification of cancer cells sensitive to the pan-Class I PI3K agent pictilisib (false-discovery rate or FDR < 10^−10^) and the dual class I PI3K/mTOR drug GDC-0980 (FDR < 0.0019), as well as other targeted agents in a large panel of cancer cell lines [56]. In the future, it will be crucial to test the predictive power of such an aggregate scoring system of signalling pathway deregulation incorporating proteomic and phosphoproteomic data and using patient-derived samples and clinically defined response criteria to assess if this approach can reliably identify patients who are likely to respond to PI3K inhibitors.

## Rational targeted combination strategies

With the exception of the δ-targeted drugs, because of modest clinical effects observed so far with single agent PI3K inhibitors — certainly less than is seen with BRAF, MEK, EGFR and ALK inhibitors in the corresponding vulnerable genotypes — it is important that rational combinations of PI3K inhibitors are pursued to maximize the chances of revealing their full therapeutic potential in cancer patients. Moreover, this is additionally important because the PI3K pathway is a common mechanism of resistance to multiple targeted agents, and conversely, resistance to PI3K inhibitors may also develop due to aberrant compensatory signalling through other pathways. Some currently pertinent combination strategies are described in this section.

Activating *PIK3CA* mutations are the most frequent genomic alterations in oestrogen receptor (ER)-positive breast cancers and PI3K inhibition results in increased expression of ESR1 mRNA and ER protein together with enhanced ER-driven transcriptional activity, with these effects seen in laboratory models and in tumours from patients treated with the PI3Kα-specific inhibitor BYL719 [57]. Importantly, data from preclinical models show that the anti-ER drug fulvestrant markedly increases the response of ER-positive tumours to PI3Kα inhibition, resulting in major tumour regression in *in vivo* animal models, suggesting that combined PI3K and ER inhibition is a rational approach to target such tumours.

Building on this, a randomized phase II breast cancer study (FERGI) assessing the combination of pictilisib and the ER antagonist fulvestrant in ER-positive aromatase inhibitor-resistant advanced or metastatic breast cancer was recently reported [58]. In this study, although no overall difference was observed in median progression-free survival (PFS) between the combination of pictilisib and fulvestrant versus placebo and fulvestrant, an exploratory analysis demonstrated that patients who were both ER and progesterone receptor (PR) positive were 56% less likely to have disease progression when treated with the experimental arm versus placebo (median PFS, 7.4 versus 3.7 months; *p* = 0.002).

CDK4/6 inhibition was shown to sensitise cancer cells to PI3K inhibition, producing a greater reduction of cell viability. Tumours from patients responding to the PI3Kα-specific inhibitor BYL719 demonstrated suppression of RB phosphorylation, while non-responding tumours exhibited sustained or increased levels of phospho-RB after treatment [59^•^]. This suggested persistent phosphorylated Rb post-PI3K inhibitor exposure as a potential clinical marker of acquired or intrinsic resistance to PI3K inhibition. Furthermore, the combination of PI3K and CDK4/6 inhibitors was shown to overcome intrinsic and adaptive PI3K inhibitor resistance, causing regressions in *PIK3CA* mutant tumour xenograft models. Clinical studies are underway with the combination of CDK4/6 and PI3K inhibitors [60^••^].

Resistance to PI3K inhibitors has been observed in cancer cells in which upstream signalling is increased via upregulated secretion of the EGFR ligand amphiregulin [61], increased expression of receptor tyrosine kinases (RTKs) such as EGFR, HER3 and IGF1R/IR due to FOXO-mediated transcriptional upregulation [62], and activated NOTCH signalling [63]. Promising preclinical data generated from studies combining RTK and PI3K inhibitors indicate that antitumour responses to PI3K inhibition can be significantly enhanced in triple negative breast cancer models through the combination with a dual EGFR/HER3 antagonist *in vitro* and *in vivo*, with important implications for this relatively hard to treat subtype [64]. Overexpression of kinases RSK3 and RSK4 has also been demonstrated to confer resistance to PI3K inhibitors through the attenuation of apoptosis and upregulation of protein translation [65].

Other resistance mechanisms to PI3K inhibitors involving MYC oncogene activation have also been proposed. A chemical genetic screen in breast cancer cell lines implicated the activation of both MYC and Notch pathways as putative resistance mechanisms to PI3K inhibitors [63]. Use of an engineered mouse model with an activated *PIK3CA* H1047R allele showed that MYC activation resulted in acquired resistance to specific PI3K inhibitors, independent of the PI3K pathway [66], while a study of genetically defined mammary epithelial cells also implicated either MYC or eukaryotic translation initiation factor 4E (eIF4E) amplification, both associated with elevated 5′ cap-dependent protein translation, as a resistance mechanism to the dual PI3K/mTOR inhibitor BEZ235 [67]. This indicates that drivers of protein translation confer resistance to PI3K pathway-targeted drugs.

The preclinical combination of PI3Kα inhibitors with EGFR, AXL, or PKC inhibitors can overcome the resistance observed in squamous cell carcinomas (SCC) of the head and neck, where SCC cells that become resistant to PI3Kα inhibition maintain PI3K-independent activation of mTOR [68].

The oncogenic RAS–RAF–MEK–ERK MAPK pathway is possibly the most commonly reported escape mechanism following PI3K pathway inhibition because of signalling crosstalk [35, 69]. While dual inhibition of PI3K and MAPK signalling has been reported to be synergistic for increased cell death in preclinical models, data also suggest that narrow therapeutic indices due to overlapping toxicities may be a challenge to the clinical success of this combination, unless intermittent dosing strategies prove efficacious [70]. For example, during the Phase I study of the combination of the PI3K/mTOR inhibitor SAR245409 (Merck Serono) and MEK inhibitor pimasertib, escalation to their single agent doses was precluded because of dose-limiting toxicities and the requirement for dose modifications with chronic dosing [71]. A phase Ib trial of buparlisib and the MEK1/2 inhibitor trametinib (GSK) showed promising antitumour activity in patients with *KRAS* mutant ovarian cancer; however, the chronic tolerability of the combination at the recommended phase II dose was challenging, due to frequent dose interruptions and reductions for toxicity [72].

A recent preclinical study showed that baseline PI3K activation is associated with greater resistance to inhibitors of poly-ADP ribose polymerase (PARP) in small cell lung cancer [73]. Previous studies had demonstrated synergy between PARP and PI3K inhibitors in BRCA1-deficient mouse models [74] and shown that PI3K blockade promotes homologous recombination deficiency by downregulating BRCA, thereby sensitising BRCA-proficient tumours to PARP inhibition [75]. Another study supported the combinatorial inhibition of PARP and PI3K as an effective option for PTEN-deficient prostate cancer [76]. Clinical trials have recently begun to test this rational combination hypothesis in cancer patients [25].

## Future perspectives and conclusion

Together with the approval of the PI3Kδ inhibitor idelalisib for different B-cell malignancies, there is now a large armamentarium of different PI3K inhibitors with diverse isoform-selectivity profiles in early to late clinical trial testing, involving both monotherapy and combination strategies. Learning from the success with the PI3Kδ inhibitors and noting the modest clinical benefit to date with single agent administration of other PI3K drug classes, it is clear that we need to refine our views both of target involvement and the corresponding therapeutic strategies. Rational patient selection through the use of analytically validated and clinically qualified predictive biomarkers will be crucial. Obtaining tumour biopsies for molecular profiling as well as minimally invasive analysis, such as use of circulating plasma DNA, will be important to predict sensitive and resistant patients, with further refinement to identify clonal involvement to guide treatment selection [77^•^].

Of likely importance is a very recent report that — in contrast to mutations in the RAS–MEK signalling axis that were less subclonal — 15% of mutations in genes in the PI3K–AKT–mTOR signalling axis across different tumour types are subclonal rather than truncal [49]. Responses are likely to be less impressive and durable where oncogenic mutations are subclonal rather than truncal.

Where single agent PI3K inhibitor activity is not likely to be effective owing to factors such as the lack of appropriate genomic aberrations, the presence of clonal heterogeneity or the development of adaptive feedback loops, it will be essential to combine PI3K inhibitors — either with other molecularly targeted drugs, hormonal agents, or alternatively cytotoxic chemotherapy — in order to fully reveal their full therapeutic potential. Such advances will only be possible through further improvements in our understanding of the underlying biology of tumours and the development of appropriate isoform-selective drugs in specific molecular settings. It is likely that there will continue to be unexpected surprises. For example, amplification of the mutant *PIK3CA* locus has been demonstrated to produce resistance to PI3K inhibition [78].

Thereafter, we will need to identify the best PI3K inhibitor combinations and to prioritize for clinical evaluation those with the highest likelihood of patient benefit and minimal toxicity. In the future, as preclinical models and molecular profiling technologies become increasingly sophisticated and robust, it is likely that we will have the necessary biomarker tools and knowledge to support clinical combination trials. For example, patient-derived models of acquired resistance have recently been shown in promising early studies to enable identification of effective drug combinations for use in patients with non-small cell lung cancer [79^••^]. Ultimately, combination studies involving PI3K inhibitors may be limited by toxicity, and it will therefore be important to explore different dosing regimens, including pulsatile schedules, which may improve tolerability and optimise antitumour activity. With such intermittent schedules, it may even be possible to combine multiple drugs akin to approaches used successfully to combat drug resistance in infections such as HIV.

In conclusion, these are exciting times in precision cancer medicine, with a range of different PI3K inhibitors available for use either as monotherapy or in combination regimens. We now need to refine the clinical application of PI3K inhibitors with different isoform selectivities using robust predictive biomarkers and rational combinatorial use with other antitumour agents, so as to maximally impact cancer and provide patients with the greatest chance of valuable benefit.

## Conflict of interest

TA Yap, L Bjerke, PA Clarke and P Workman are current employees of The Institute of Cancer Research, London, which has a commercial interest in the development of PI3K inhibitors, including pictilisib, and operates a rewards-to-discoverers scheme. TA Yap has received research funding from AstraZeneca. P Workman has received research funding from Astellas Pharma and Piramed Pharma; has ownership interest in Chroma Therapeutics and previously Piramed Pharma; and is or was a consultant/advisory board member for Chroma Therapeutics, Nextech Invest, NuEvolution and Piramed Pharma.

## References and recommended reading

Papers of particular interest, published within the period of review, have been highlighted as:

• of special interest
•• of outstanding interest
